# Use of MRI prior to corticosteroid injections of wrist joints in children with JIA

**DOI:** 10.1186/1546-0096-10-S1-A37

**Published:** 2012-07-13

**Authors:** Alaina M Davis, JH Kan, T Brent Graham

**Affiliations:** 1 Vanderbilt University, Nashville, TN, USA

## Purpose

The use of intra-articular corticosteroids in children with juvenile idiopathic arthritis (JIA) is an accepted therapeutic option as their use has been found to be safe and proven to facilitate rapid resolution of signs and symptoms of inflammation. An increasing number of studies have described improved sensitivity of magnetic resonance imaging (MRI) compared with conventional radiography in detection of early synovitis, tendinopathy, bone edema, and bone erosions. The purpose of this study was to document joints within the wrist and carpus with active synovitis in children with JIA using MRI and to explore how MRI localization of affected joints prior to intra-articular corticosteroid injection affects outcomes.

## Methods

A retrospective chart review was completed to identify 26 patients who received a total of 37 fluoroscopic guided corticosteroid wrist injections between the years 2009-2010. Nine of the patients identified underwent wrist MRI prior to the procedure (group A). Seventeen patients (group B) did not have MRI performed prior to injection. In group A, MRI showed evidence of inflammation within 7 radiocarpal joints, 9 midcarpal joints, 5 radioulnar joints, 3 first carpometacarpal joints, and 5 second through fifth carpometacarpal joints. Joint injections were performed to target these joints in group A patients. In group B, the radiocarpal joint was injected in all cases except for 4 patients in whom the midcarpal joint was injected and 1 patient whom also had the first carpometacarpal joint injected.

## Results

All patients in group A (n=9) maintained or decreased therapy at follow-up appointment. However, 4 of the 17 patients in group B required escalation of therapy at follow-up appointment. Groups did not differ in likelihood of escalation of therapy (Fisher’s exact test, p=0.26), likely due to the limitation of small sample size. Erosions were documented in 6 midcarpal and 4 second through fifth carpometacarpal joints but no radiocarpal or radioulnar joints.

## Conclusion

This study proves there are multiple affected joints within the wrist and carpus of children with JIA. Furthermore, it suggests that performing MRI prior to intra-articular corticosteroid injection to identify affected joints compared to clinical exam alone may help better target therapy and improve outcomes. More extensive research is needed to answer this question definitively.

## Disclosure

Alaina M. Davis: None; J.H. Kan: None; T. Brent Graham: None.

